# Impacts of human-related practices on *Ommatissus lybicus* infestations of date palm in Oman

**DOI:** 10.1371/journal.pone.0171103

**Published:** 2017-02-06

**Authors:** Khalifa M. Al-Kindi, Paul Kwan, Nigel R. Andrew, Mitchell Welch

**Affiliations:** 1 School of Science and Technology, University of New England, Armidale, New South Wales, Australia; 2 Centre for Behavioural and Physiological Ecology, Zoology, University of New England, Armidale, New South Wales, Australia; University of Vigo, SPAIN

## Abstract

Date palm cultivation is economically important in the Sultanate of Oman, with significant financial investments coming from both the government and private individuals. However, a widespread Dubas bug (DB) (*Ommatissus lybicus* Bergevin) infestation has impacted regions including the Middle East, North Africa, Southeast Russia, and Spain, resulting in widespread damages to date palms. In this study, techniques in spatial statistics including ordinary least squares (OLS), geographically weighted regression (GRW), and exploratory regression (ER) were applied to (a) model the correlation between DB infestations and human-related practices that include irrigation methods, row spacing, palm tree density, and management of undercover and intercropped vegetation, and (b) predict the locations of future DB infestations in northern Oman. Firstly, we extracted row spacing and palm tree density information from remote sensed satellite images. Secondly, we collected data on irrigation practices and management by using a simple questionnaire, augmented with spatial data. Thirdly, we conducted our statistical analyses using all possible combinations of values over a given set of candidate variables using the chosen predictive modelling and regression techniques. Lastly, we identified the combination of human-related practices that are most conducive to the survival and spread of DB. Our results show that there was a strong correlation between DB infestations and several human-related practices parameters (*R*^2^ = 0.70). Variables including palm tree density, spacing between trees (less than 5 x 5 m), insecticide application, date palm and farm service (pruning, dethroning, remove weeds, and thinning), irrigation systems, offshoots removal, fertilisation and labour (non-educated) issues, were all found to significantly influence the degree of DB infestations. This study is expected to help reduce the extent and cost of aerial and ground sprayings, while facilitating the allocation of date palm plantations. An integrated pest management (IPM) system monitoring DB infestations, driven by GIS and remote sensed data collections and spatial statistical models, will allow for an effective DB management program in Oman. This will in turn ensure the competitiveness of Oman in the global date fruits market and help preserve national yields.

## 1. Introduction

Date palm (*Phoenix dactylifera*) cultivation is economically important in the Sultanate of Oman, with significant financial investments coming from both the government and private individuals. However, a widespread Dubas bug (*Ommatissus lybicus* Bergevin) infestation has impacted regions including the Middle East, North Africa, Southeast Russia and Spain, resulting in substantial damage to date palms [1–6]. Dubas bugs are yellowish green in colour; females range in length from 5 to 6 mm, and males range from 3 to 3.5 mm. Male and female bugs are primarily distinguished by a spot on the females. Moreover, male bugs have a tapered abdomen and larger wings relative to the females. Nymphs have five instars and each instar has waxy filaments. However, the nature and level of DB infestation vary with location, conditions and human-related practices.

Two generations of Dubas bugs appear yearly in Oman, one in spring and another in autumn [7]. In the spring generation, eggs start hatching from February to April where nymphs pass through 5 instars to become adults in approximately 6–7 weeks. The eggs aestivate during the hot season (i.e., summer) until the autumn generation where they start hatching from late August to the last week of October. A nymph takes about 6 weeks to develop into an adult, which lives for about 12 weeks. Each female can produce more than 120 eggs, which are laid by insertion into holes in the tissue of date palm fronds at the end of each season [8].

DBs are dynamic on leaflets, rachis, fruiting bunches and spines during different stages of the date palm lifecycle. They cause direct and indirect damage to palm trees. The direct damage arises once the nymphs and adults feed by sucking sap from leaflets and rachis in the spring and autumn generations. The indirect damage comes from the deterioration of palm fruits and other crops that are cultivated underneath the palm trees through honeydew, which attracts dust, dry leaflets and rot fungi. The copious feeding of DBs weakens the tree, whereas the honeydew pollutes the vegetation and provides a substrate for the growth of black sooty mould that reduces photosynthesis of the frond surface, which then becomes chlorotic after several months [7,9,10].

DBs require much effort and money for control in several countries in the world, including the Sultanate of Oman [11]. The impacts of DBs are more severe than other insect pests, like red palm weevil [12,13]. It is the primary cause of infestation that leads to death of palm trees. In Oman, the control activities that aim to exterminate or reduce DB infestations have concentrated on the use of insecticides that include both ground and aerial sprays. Given the significant economic impact of this pest, research into effective management strategies demands high priority. Several insecticides have been evaluated for DB control in Oman; SUMI-ALPHA 5 EC is effective as a ground spray and KARATE 2 ULV, TREBON 30 ULV and SUMICOMBI 50 ULV have achieved some measure of success as aerial sprays [14,15]. KARATE-ZEON was also found to be very effective because it gave a 100% reduction in the numbers of DB instars and adults between three and seven days after application.

However, the use of the most effective pesticides is restricted because of their side effects such as irritation [14]. In Israel, systemic carbamates (e.g., aldicarb and butocarboxim) have been used successfully, whereas in Iraq, dichlorvos (DDVP) injected directly into infected palms has been successful in suppressing the pest population [3]. However, these methods are expensive and can have negative environmental impacts on both non-target species, particularly the natural enemies of the DB *(e*.*g*., *Aprostocetus sp*., *Oligosita sp*., *and Runcinia sp*.*)*, and on human health [16,17]. Research has shown that some pesticide residues can persist on date fruits for up to 60 days after application [18–20]. Moreover, chemical control measures have met with limited success in Oman, where DBs continue to pose a major challenge to the agricultural industry.

Although there are a number of studies on the biology and ecology of DBs, applications of geographic information systems (GIS), remote sensing (RS) and spatial analysis of DB infestations in the Sultanate of Oman are limited [21]. In fact, there is no focused research on the spatial distribution and modelling of the DB and its relationships with human-related factors. Therefore, it became necessary to conduct a survey on DBs in different regions and conditions within the Sultanate to enable development of an applied research program that aims to decrease losses caused by DBs as well as to suggest an effective IPM program [22]. We highlighted the importance of understanding and regulating date palm human-related practices for successful DB management. This study was conducted in date cultivations in the nine governorates of northern Sultanate of Oman to ascertain the impact and to quantify the influence of human-related practices adopted by farmers on the infestation levels of DB on date palms.

The main objective of this study is to apply spatial analytic and modelling techniques to gain an understanding of the correlations between various human factors related to date palm farming as well as the distribution and density of DBs. To achieve this, we considered the contributions of factors including irrigation type, planting (row spacing), pruning, removing or keeping suckers, insecticides, fertilising, tree density per hectare, removing unproductive palms, weeds, field crops, cultivation interfaces, educated and non-educated issues. The study addressed this question: “What are the relationships between the observed patterns of DB infestation and human-related practices (i.e. variables)?”

## 2. Methods and materials

### 2.1 Study area

**Fig 1:**
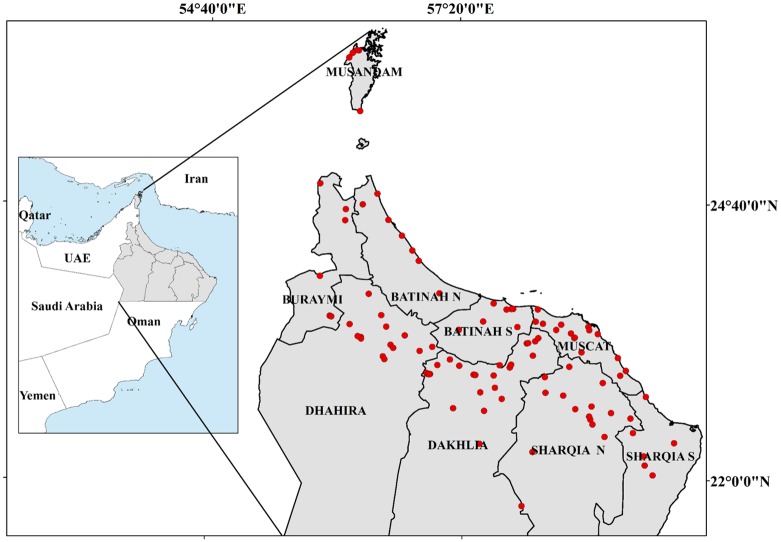
The study areas (nine governorates in the northern Sultanate of Oman) with sampled date palm plantations highlighted in red (Esri ArcGISTM 10.3).

Significant numbers of palm trees are primarily grown in the northern part of Oman, where agriculture and climate conditions satisfy the requirements of production [23]. The date palm has the ability to survive under the adverse conditions found there [24]. Although the study area covered 9 governorates in northern Oman, analysis of our data revealed that most commercially managed and high-quality data palm plantations are located in 6 of these governorates: Al-Dakhliyah (Samail), Al-Dhahirah (Ibri), Al-Batinah (North and South), and Al-Sharqiyah (North and South) (see Fig 1). In addition, Northern Oman (26°50N to 22°26N, and 55°50’E to 59°50E) has also experienced high infestations [25] (Fig 1).

### 2.2 Dependent and independent variables

The spatial correlations between DB infestation (dependent variable) and human-related or cultural practices (candidate/independent variables) such as irrigation type, row spacing (distance between trees), palm density per hectare, palm and farm services (pruning, old tree removal, remove weeds), offshoots removal from mother palm, cultivation interfaces, fertilisation, educated and non-educated employees are critical. There are a number of methods that can be used to determine the spatial correlations. Four commonly used spatial regression techniques include exploratory regression analysis (ERA), geographically weighted regression (GWR), ordinary least squares (OLS) and logistic regression (LR). However, finding a good spatial regression model can be complicated, particularly when researchers or users have an extended list of potential explanatory factors that support their analyses [26].

In this study, ERA was chosen to investigate the spatial correlation between DB infestation and the human-related practice variables mentioned earlier. ERA is a statistical data mining tool that can be used to determine properly specified OLS models. ERA builds OLS models using all possible combinations of a given list of candidate explanatory variables and assesses which, if any, satisfy the essential OLS criteria. The regression analysis applies the OLS method to perform diagnostic tests based on the joint Wald statistic, Koenker (BP) statistic, and Jarque-Bera statistic [27,28]. Exploratory spatial data analysis (ESDA) tools create a report summary comparing all the satisfied models, which helps users identify models that do not pass and thus provides useful information to determine the problem areas. The significance of each variable is given as the percentage of all models tested for which the candidate explanatory variable was statistically significant [26]. OLS can be computed by:

$$Y=\beta_{0}+\beta_{1}\times X_{1}+\beta_{2}X_{2}+\ldots \beta_{n}X_{n}+\varepsilon$$ (1)

where Y is the dependent variable (number of nymph per leaflets), *X*_1_ + *X*_2_…, *X*_*n*_ represent the independent variables (explanatory variables), *β*_*1*_ *+β*_*2*_*…*, *β*_*n*_ are regression coefficient, and ɛ is residual.

### 2.3 Geographically weighted regression

Geographically weighted regression (GWR) was used to create coefficient maps, residuals, local R-squared values, condition numbers and predictions. GWR performs a local form of linear regression that can be used to model spatially varying relationships [29]. A GWR model has been used to detect high-risk infestations caused by mountain pine beetle invasions of lodge-pole pine forests over large areas [30]. However, GWR has not consistently differentiated between stationary and nonstationary data generating processes. Moreover, multicollinearity-estimated coefficients may bias the results, and it is unclear which tests can reliably diagnose model problems [31].

### 2.4 Data collection process

There are two methods that have been commonly used to identify the economic threshold of DB infestation during the autumn and spring generations. The first method involves counting the number of nymphs on each leaflet. The Ministry of Agricultural and Fisheries (MAF) in Oman considered a scale from low, moderate to heavy infestation. Low infestations are cases where five or less nymphs (instars) are found on the leaflet; moderate infestations are cases where 5 to 10 nymphs are found per leaflet; heavy infestations are cases where there are 10 or more nymphs per leaflet [11]. The second method involves determining the infestation level by counting the number of honeydew droplets from palm trees [7].

In this study, we have selected the counting of nymphs method. We modified the MAF’s suggestion slightly and classified the DB infestation levels into 4 groups (no infestation, low, moderate and high) in ArcGIS 10.3 as follows: no infestation (no nymphs per leaflet); low infestation (1 to 5 nymphs per leaflet); medium infestation (6 to 9 nymphs per leaflet); and high infestation (10 or more nymphs per leaflet). The number of nymphs per leaflet at each potentially infested site was collected in only the autumn generation in 2015.

This survey covered two levels of palm, small and medium trees, respectively. We selected 3–5 point locations (Easting and Northing) in each district and different locations were considered, such as farms close to sea, desert or mountain plain that covers the nine governorate of northern Oman.

The irrigation type and the spatial data were obtained from the Ministry of Regional Municipalities and Water Resources (MRMAWR) of the Sultanate of Oman in December 2015 and combined with questionnaires to gain more accurate details (see Table 1). After measuring the distance between trees, we decided on three categories to indicate the amount of row spacing between trees: low if the distance is between 1 to 3 m, medium from 4 to 7 m, and high from 8 to 10 m. The density of palm trees in each farm was determined by calculating the number of palm trees per hectare. The IKONOS satellite images for 2015 (5 m spatial resolution) for the study area, used with permission from the National Survey Authority (NSA) of the Sultanate of Oman, were processed by the image segmentation (IS) functions available in the software ENVI 5.1 to extract the density information of palm canopies. The crown of palm canopies was used as the proxy indicator to calculate the real tree density. Sample locations were identified from the satellite image by examining the Normalised Difference Vegetation Index (NDVI).

**Table 1:** Independent variables and the dependent variable and their classification index levels.

Independent variables	Index values			
	0/No	1/Yes	2	3
Row spacing (distance between trees)	-	1–3 m	4–7 m	8–10 m
Palm density (trees per /hectare)	-	>100	50–100	1–49
Group 1: Farm service (indicates 5 elements)	-	≤ 2	3 out of 5	5 out of 5
Pruning regime Remove weeds Debris regime Thinning regime Remove old tree (non-economic palms)				
Group 2: Removal offshoots	No	Yes	-	-
Group 3: Pesticides	No	Yes	-	-
Irrigation system		Flood [24]	Borehole	Drip
Fertilisation	No	Manure	Urea	-
Field crop	No	Maize	Alfalfa	-
Cultivation interface (grass)	No	Yes		
Educated	No	Yes	-	-
Dependent variable	Index values			
DB infestation levels (Number of nymphs per/leaflets)	0	≤ 5	6-9	≥10
	No	Low	Medium	High

In this study, NDVI served a surrogate measure of palm plantation density and homogeneity in the neighbourhood surrounding an image pixel. The degree of palm plantation density is expressed in the image information in percentages: high palm plantation canopy density (more than 100 palms per hectare), medium palm plantation canopy density (palms from 50 to 100 per hectare) and low palm plantation canopy density (palms from 0 to 49 per hectare). Large area would be expected to result from high density palm (deciduous tree canopy) or more than 100 palm trees per /hectare. Thus, areas containing a higher biomass and more reflectance leaf area will have a higher NDVI value. NDVI can be computed as NDVI = (NIR-RED) / (NIR+RED) [32], where NIR is the recorded radiance in the near infrared, and RED is recorded value in the red portion of the spectrum for a particular image pixel. The NDVI for non-vegetation area are negative values, while vegetated areas have values between 0.1 and 1.0.

The questionnaire data on number of trees/hectare are used as ground truth data to link satellite image information to the quality of tree plantations. In the absence of available detailed information for measuring the palm density for the study areas, we believe that the approach described would best approximate the reality on the ground at local scales.

The questionnaire data on number of trees/hectare are used as ground truth data to link satellite image information to the quality of tree plantations. In the absence of available detailed information for measuring the palm density for the study areas, we believe that the approach would best approximate the reality on the ground at local scales. This analysis was used for broad scale application and was conducted much cheaper and more effective using remotely sensed imagery than with extensive field based surveys. We determined the criteria used for classification of palm density per hectare for each sampling.

We divided the services (i.e., palm and farm services) to prevent DB infestation into three groups (or factors). The first group includes measures such as pruning, removing unproductive palms, removing weeds under trees, debris removal and thinning regime. If these services are performed regularly, this factor are given the number 3, which means that the palms and farm are in a high service condition; similarly, the number 2 means medium service, and the number 1 means low service. The second group of services includes removing the offshoots from the mother palm. The third group is pesticide application; we collected insecticide data from the MFA in 2015 and then combined them with our field survey for a more detailed investigation and more accurate results (see Table 1). The survey covered 185 fields (observations and questionnaire) collected in the autumn DB generation in 2015.

The fertilisation factor was also surveyed; the questionnaire investigated the type of fertilisation used regularly on the farm, the employees hired (educated and non-educated), the cultivation interface (grass), and field crops (alfalfa and maize) as explanatory variables (see Table 1).

The kernel was specified as a fixed distance to solve each regression analysis. The bandwidth was specified using the AIC (ESRI, 2015) to determine the extent of the kernel. This was the bandwidth or number of neighbours used for each sample’s number of estimation and was perhaps the most important parameter for ERA because it controlled the degree of smoothing in the model.

## 3. Results

### 3.1. Exploratory regression tools, OLS and GWR findings

The model variables that best explain the occurrence of DB in the study area, along with their variance inflation factors (VIF) are shown in Table 2. Variance Inflation Factors (VIF) is a test designed to measure if two or more variables are telling the same story (i.e. collinearity) [33–35]. The idea is that any variable that has a value of greater than 7.5 should be removed from consideration. As the initial steps in the OLS regression analysis, multicollinearity testing by variance (VIF) was run on the pool of 15 independent variables, selected to correspond to the conceptual model. For the 49 regressions, the average VIF value is 1.96, with the minimum value of 1.18. The VIF values are all well under the ESRI-defined threshold of 7.5, which confirmed that these variables are not redundant. The maximum was (flood irrigation) with 4.12, which stabilizes model multicollinearity.

**Table 2:** The best fit model variables from OLS exploratory regression and their related VIF values.

Variables	VIF
**Flood (*Falaj*) irrigation**	4.12
**Borehole irrigation**	3.44
**Row spacing**	2.23
**Fertilisation (manure)**	2.08
**Fertilisation (urea)**	2.04
**Group 2: Offshoots removal**	2.02
**Group 1: (pruning, remove weeds, remove unproductive palm, thinning and debris regime)**	1.98
**Maize grown in the same farm**	1.78
**Group 3: % Pest control (pesticides)**	1.71
**Grass under trees**	1.56
**Alfalfa**	1.46
**Density palm (trees per hectare)**	1.31
**% Educated employees**	1.28
**% Non-educated employees**	1.23
**Drip irrigation**	1.18

**Fig 2:**
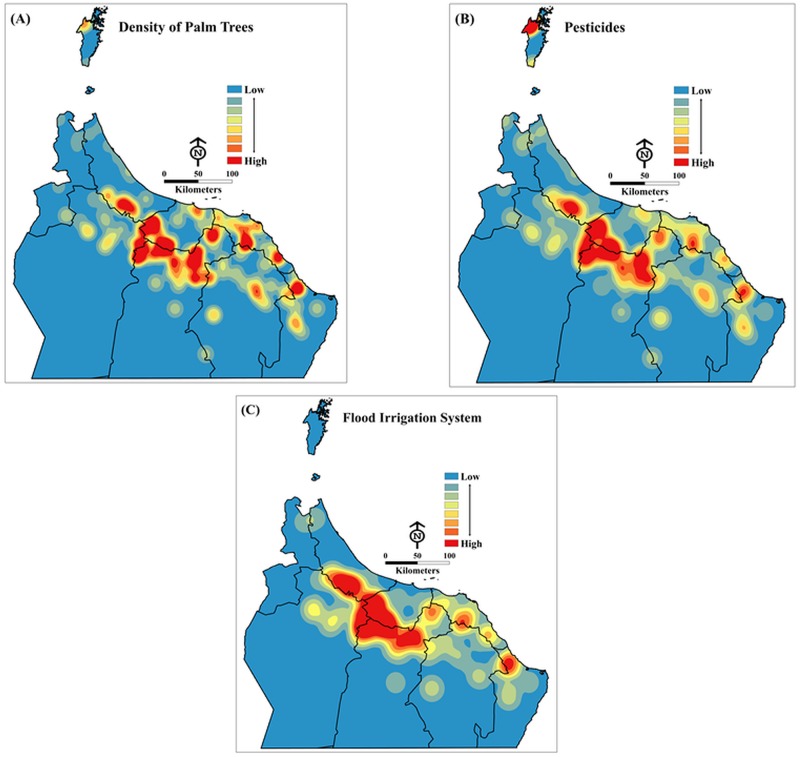
Example of GWR parameters (βs) for (A) density of palm trees per acre, (B) pesticides and (C) flood irrigation system. The examples show how modelled relationships vary across the study area. All maps are of the same scale (Esri ArcGISTM 10.3).

The significance values were also valuable in removing variables from subsequent modelling attempts. Although the visible clustering indicated that the amount of impervious surface may have been a predicting feature (see Fig 2), with respect to model importance it was among the least likely predictors. Instead, distance between trees, palm tree density, pesticides and date palm and farm services (pruning, thinning, remove weed, remove unproductive trees, debris removal regime) were the four most significant explanatory variables and thus appeared in the majority of passed models (see Table 3).

**Table 3:** Explanatory regression model variables and the percentage of prototypes in which were found significant.

Variable	% Significant
**- Row spacing (distance between trees)**	100
**- Density tree per hectare**	99.88
**- Pesticides**	99.58
**- Date palm and farm services (pruning, thinning, remove weed, remove unproductive trees, debris removal regime).**	99.5
**- Flood (falaj) irrigation system**	84.11
**- Fertilisation (manure)**	82.43
**- Offshoots not removed from the mother palm tree.**	81.31
**- Non-educated**	43
**-Borehole irrigation system**	23.74
**-Fertilisation (Urea)**	21.43
**-Educated**	19.02
**-Planting maize crop**	11.93
**-Drip irrigation**	10.50
**-Grass**	6.18
**-Planting alfalfa crop**	0.09

**Fig 3:**
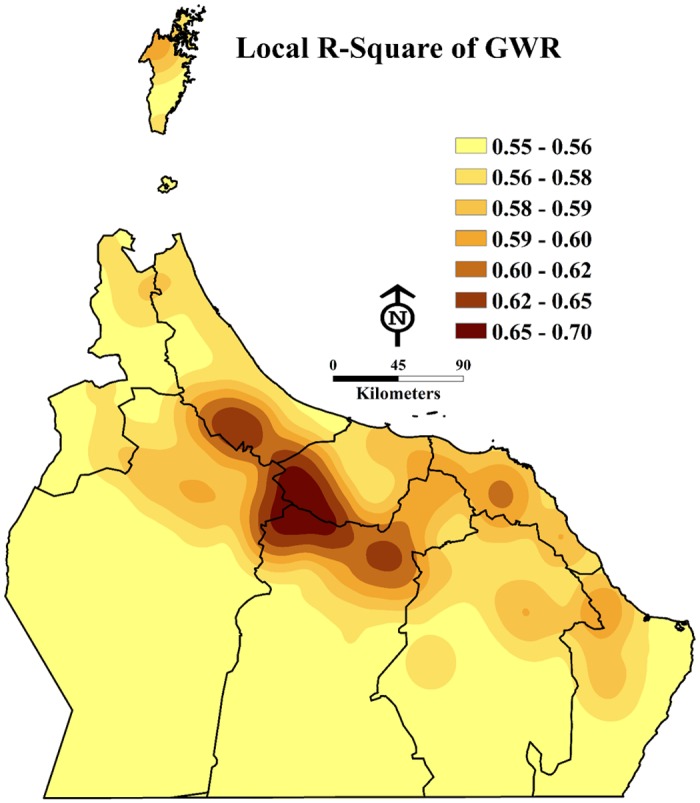
Local R2 of GWR shows where the model performed best (Esri ArcGISTM 10.3).

Eight of the fifteen variables in Table 3 above had significant p-values of less than 0.05, which indicates strong relationships between the individual explanatory variables and the dependent variable (see Table 4). It should also be noted that whereas these eight variables provide the highest adjusted R^2^ value, the pesticides factor alone met all OLS assumptions with an R^2^ value of 61% and a higher AIC value (732). Each variable added into the model improved the R^2^ percentage by approximately 2% and reduced the AIC number by 2. The coefficient values displayed in Table 4 and Fig 3 show that the percentage of row spacing between palm trees, density of palm trees per acre, pesticides, date palm and farm services, and flood (*falaj*) irrigation system have the strongest correlations with DB infestation levels and that other variables still proved to predict a strong correlation with DB infestation levels in the study area. The maps of local R^2^ in Fig 3 show where the model performs best.

**Table 4:** P-values showing statistically significant variables.

Variable	Coefficient [a]	StdError	T-Statistics	Probability [b]	Robust_t	Robust_SE	Robust_Pr [b]
**-Intercept**	15.093	5.697	2.649	0.009*	2.671	5.650	0.009*
**-Palm density**	8.445	2.089	4.043	0.000*	4.376	1.683	0.005*
**-Flood (falaj) irrigation**	2.961	1.163	2.546	0.013*	2.905	1.019	0.009*
**-Row spacing**	-2.968	1.046	-2.836	0.006*	-2.666	1.113	0.001*
**-Group [1]**	-5.367	1.164	-4.611	0.000*	-4.381	1.224	0.000*
**-Removal offshoots**	-5.936	1.589	-3.734	0.000*	-3.068	1.934	0.003*
**-Pesticides**	3.151	0.689	4.571	0.000*	4.395	0.717	0.000*
**-Non-educated**	-3.569	1.492	-2.392	0.018*	-2.615	1.365	0.010*
**-Fertilisation (manure)**	-9.784	2.682	-3.648	0.000*	-4.453	2.197	0.000*

*An asterisk next to a number indicates a statistically significant p-value (p < 0.01).

[a] Coefficient: represents the strength and type of relationships between each exploratory variable and the dependent variable.

[b] Probability and Robust Probability (Robust_ Pr): Asterisk (*) indicates a coefficient is statistically significant (p < 0.01); if Koenker

(BP) Statistic [f] is statistically significant, use the Robust Probability column (Robust_Pr) to determine coefficient significance.

Group [1]: incudes pruning, remove weed, remove non-economic palm, thinning and debris regime.

**Fig 4:**
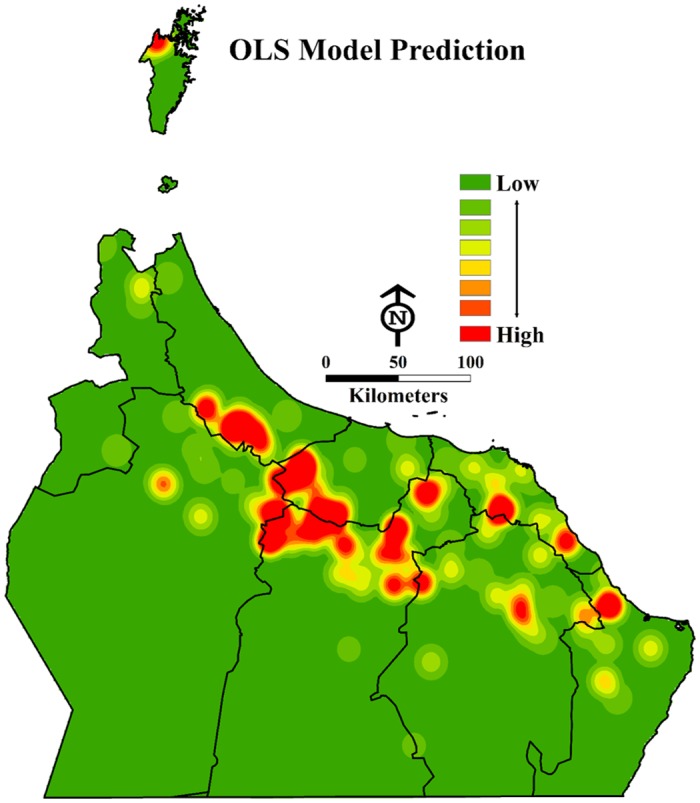
The OLS model predictions of impacts of all human-related practice variables on DB infestation on date palm plantations in the study area. The prediction model shows the areas at risk of DB based on different human-related practice parameters (Esri ArcGISTM 10.3).

A significant result of model predictions is evident in the mapping of residual standard deviation. The model produced under predictions; it is likely that other variables still proved to predict a strong correlation with DB infestation levels in the study area shown in Fig 4.

**Fig 5:**
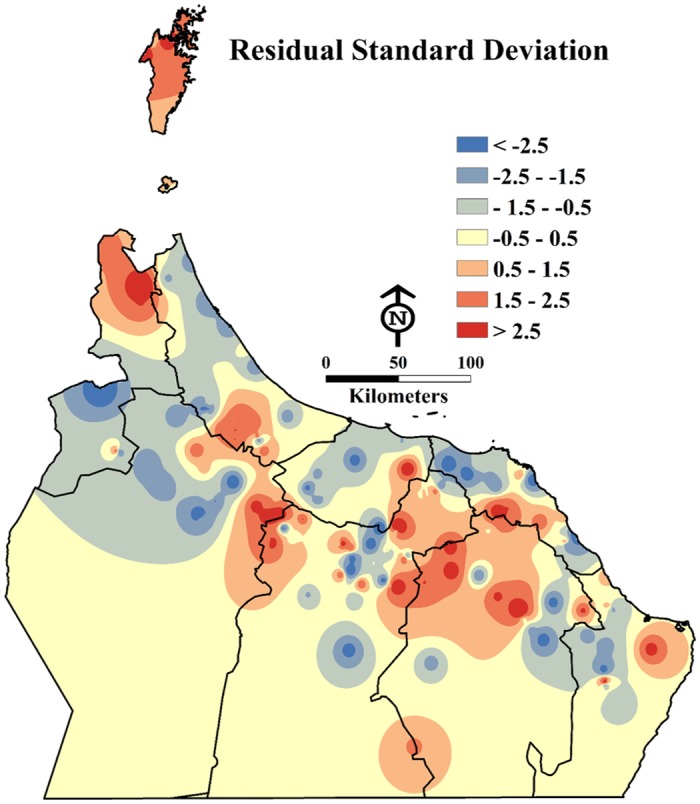
A map showing the spatial pattern of under or over predictions (in other words, lower or higher than actual infestation level) based on the calculated residual standard deviations from the model (Esri ArcGISTM 10.3).

The model explained 70% of the impacts of human-related practices on DB infestation levels. The Koenker (BP) statistic returned a p-value of 0.0029, which was highly statistically significant; this means that the model showed consistent relationships across the geographic areas of the study. The Jarque-Bera statistic of 0.3798 was not significant, which indicated a normal distribution of residuals (see Fig 5). The Akaike’s Information Criterion (AIC) was 959.47. Similar to the adjusted R-square value, the AIC is an indicator of good model presentation.

We ran the spatial autocorrelation tool on the residual standard deviations (RSD) of each area in order to investigate where the model predictors were greater or less than (over/under predictions) reality (see Fig 5). It should be noted that even the same orchards (locations) that were predicted high or low remained less than 2.5 standard deviations (SD) from the mean. For the under predictions, the largest SD was 0.9 and for the over predictions a single orchard had a 2.72 deviation, whereas the next largest was 2.06.

The final hypothesis of the OLS model requires a minimum of 0.10 for the spatial autocorrelation p-value. Given the result value of 0.56, the selected model is shown to be neither excessively clustered nor dispersed (random). The GWR tool was also used but did not improve the model in our study. However, according to the best practices for model spatial correlation, it is important to consider the use of GWR when using the OLS Exploratory Regression method [26]. The GWR model had an AIC value of over 732 and the adjusted R-square value of 0.70. However, the only difference between the OLS and GWR model was a higher AIC, which confirmed that GWR would not improve the model.

## 4. Discussion

The results of the OLS exploratory method revealed a model that confidently predicts 70% of the impact of DB infestation on the date palms in the study area. Realistically, a higher R^2^ value would have predicted even higher confidence in the model; however, OLS specifies a minimum of 50.13% in order to pass. This model is well above that goal. The selection model represents the best of 6,884 trials and resulted in a combination of factors that include row spacing (the distance between palm trees), palm density (palms per hectare), insecticide application, irrigation type (flood, borehole and drip), fertilisation (manure and urea), offshoots removal, intercropping (alfalfa, grass and maize) and date palm farm services (pruning, thinning, removing weeds, removing unproductive trees and clean farm practices).

Within each of these variables, it should be noted that whereas the final model reflects one variable within each category, the significance of other variables may have been more prevalent in the other models; for example, the percentage of insecticide may have been more significant than distance between trees. However, in combination with other explanatory variables, the inclusion of row spacing (distance between trees) percentage yielded the better model (see Table 3).

Our results recorded in Table 4 highlights how each significant human practice variable included in this study promotes an increase or decrease of DB population as follows. We found the palm density, the type of flood irrigation and impact of pesticides are positively significant in increasing the DB populations; however, row spacing, farm service (group 1), offshoots removal, education level of employees and the fertilisation used are negatively significant.

The highest number of DB infestation levels was recorded in the farms where palms were planted at a distance of 3 to 5 m, which is a common palm spacing in many farms surveyed in the study area. This may be because a closer spacing with high palm density is likely to retain in-grove humidity. We recommend that commercial date plantations be planted at a spacing of 10 x 10 m, accommodating 80 palms per acre. Some human-related cultural practices help in preventing DB infestations greatly. Our results found that the highest level of DB infestation was negatively correlated with the row spacing. An early study [36] reported that well-spaced palms never become infested with DB. Properly spaced palms allow for wind movement and sunlight between trees [3,37,38].

Our results also found that offshoots not removed from the mother palm tree and farm services (unremoved unproductive palms, non-pruning, non-thinning and cleanliness) increased DB infestation levels in many areas of the study [39,40]. Moreover, unremoved offshoots mean that mother palms must compete to obtain enough of the important elements nitrogen, magnesium, potassium, sulphur and calcium. Iron, manganese, zinc, copper and molybdenum are also needed in small amounts. All of these elements serve as medicine for date palms for protection from diseases and infestations. In view of the severity of DB infestation on date palms in the region, we propose that human-related cultural practices that cause damage to the date palm tissue, such as shaving of fronds and removal of offshoots, be performed when DB activity is the lowest, not during the peak of the spring and autumn generations. Human-related practice controls can also be accomplished by removing old and infested fronds and by burning them, taking good care of the date palm and fronds and giving attention to agricultural cleaning.

We also found that 84.11% of the infestations in the study areas were recorded in plantations that were irrigated with flood irrigation systems, compared with 23.74% and 10.50% infestation in plantations that were irrigated through open basins or boreholes and drip lines, respectively. Open flood irrigation and high soil moisture is known to favour in-grove habitats for DB. Consequently, using a drip irrigation system with appropriate use of water is better than irrigation with bubblers because the farmer increases the efficiency of water use with minimal losses.

In view of our findings, we propose to reduce excess moisture levels in date plantations in the study area by adapting the recommended spacing at planting and using drip lines for irrigation, which would not generate excess in-grove humidity and soil moisture and would conserve the precious irrigation water resource. To date, palm trees have the capacity to survive under relatively harsh climatic and soil conditions [14] while maintaining those farms without irrigation from four to seven weeks or more before Dubas females begin to insert eggs into the fronds of the palm trees. This process diversity helps to reduce the humidity in the farms and the juice that DB is likely to sap from palm fronds and leaves [41]. Moreover, the reduced amount of irrigation may be useful to reduce the high pressure due to rich water, which assists DBs in sucking water from fronds for survival.

In Oman, although socio-economic changes have had negative effects on traditional date plantations, they have not led to the disappearance of the date palm crop. Date palm dominates agricultural production in Oman [42]. It is the traditional national staple and was Oman’s main source of income before oil, and the same applies to most Arab countries [43]. However, Oman has undergone some drastic changes that have impacted consumption patterns. For example, socio-economic changes include improvement in living standards, continuous urban drift and the introduction of technology. Al-Farsi et al. [44], reported that human-related cultural practices and post-harvest handling had a great impact on palm fruit nutritional quality.

These changes might also result from introducing non-educated labourers who lacked experience in working on farms of date palms, especially in dealing with traditional crops such as palm trees. They should also be able to take part in the training of farmers in the different components of DB control, including the knowledge of the life cycles of serious pests such as DB. This can be resolved through certain economic incentives to farming communities to preserve traditional agriculture practices such as providing jobs with satisfactory compensation for Omanis to work in palm plantations.

Successful control of DB infestations of date palms depends to a great extent on the standards of the agricultural extension services. It is the responsibility of the extension officers, who should be well-trained themselves, to train farmers and steer them away from the continuous use of chemical insecticides towards safer and more efficient alternative agents of control. Moreover, in different countries such as those of the Middle East, there could be a cadre of plant protection officers whose main duty is to carry out surveys to assess DB infestations of date palm, forward unidentified arthropod pests such as DB to identification centres, and access to identification laboratories for the isolation of fungi.

Pesticides should rarely be used for the control of DB or any pests because they also kill the natural enemies of the pests, whereas this pest is protected by its scales. Enhancements in human-related practices, such as the correct use of pheromone and light traps, proper handling of organic manures in the farms and farm hygiene, are all important measures in the reduction, elimination and control of DB infestations. Insecticide application (i.e., aerial spraying), which has been used in Oman since 1997, is different in the study region than in other areas because reduction in the amount of DB infestations on palm trees in the open plains is relatively easy, whereas this process (i.e., aerial spraying using helicopters) faces many difficulties in farms located between mountains due to elevation, slope and aspect factors.

Although presently under chemical control, DBs are a threat to date palm production in the Sultanate of Oman. In Oman, there is increasing interest in biopesticides and safe natural enemies of DB. For example, the egg parasitoids *Pseudoligosita babylnoica* (Hymenoptera: Trichogrammatidae) have been recorded in some locations and can be considered as a potential biological control agent for DBs [36]. Generally, such natural products may be highly effective but require extensive knowledge of target DB infestation and well-timed and thorough application techniques. Additionally, selection of insecticides to control DB in the date palm agro-ecosystem should be carefully done to avoid the harmful effects of these chemicals on predators and parasitoids. We believe that lack of efficient integrates pest management (IPM) and lack of efficient communications within the frame of integrates crop management (ICM) have resulted in spread of pests and diseases in different regions worldwide, including Oman.

GIS and remote sensing (RS) can be used to study the distribution and density of DB and the locations of its natural enemies. For example, remote sensing technology such as ancillary data (DGPS) can be used to collect spatial densities of the natural enemies against Dubas bug infestations. The DGPS data then can be applied to identify and visualise the hot-spot, cold spot, spatial patterns (dispersed or clustered) of these natural enemies [45]. RS tools can also be used to develop early detection for DB and study the health of date plantations. Early detection can play a crucial role in the management of DB infestation, and further research into techniques for early detection merits urgent attention. For example, Quick-bird remotely sensed images (panchromatic, hyperspectral and multispectral) and the new band World-View (Red-Edge ~705–745 nm) images can be used to map the spatial distribution of the honeydew resulting from DB infestation [22]. This approach suggests using RS to calculate the honeydew produced by DB as an indicator to estimate a threshold. The idea is based on considering the number of nymphs that can produce honeydew that cover the upper surfaces of leaflets at the critical level of infestation. Passive remote sensing methods, based on collecting and counting honeydew droplets produced by the DB, can also be used to detect and determine the effectiveness of control measures for DB. This method is effective, rapid and less hazardous and saves labour and time. Therefore, field efficacy has to be demonstrated and appropriate support and guidance transferred to the farming community.

In Oman, date palm growers manually apply fertilisers, specifically animal manure, two times per year, whereas chemicals are not commonly used in date groves. Moreover, date palms are intercropped with legumes that are used as fodder. This may make adequate nitrogen available to date palms. Al-Kharusi et al. [46] reported that the mineral fertilisers have been a significant influence on date palm quality. Ploughing the soil around the palm trunk and inspecting offshoots before planting in new groves is considered one of the best methods to restrict the spread and outbreak of DB. We propose the use of GIS tools to improve the distribution of manure at the local and field scales as a means to minimise environmental contaminations.

## Conclusions

This study has determined that many human-related cultural practices adopted in date plantations significantly impact infestation levels of DB on date palms, which should be suitably modified in order to reduce critical infestation levels. DB inhabits certain areas because those areas have suitable breeding and survival conditions. For every single plant and animal species, organisms inhabit sites that are most suitable for their needs, including DB.

A list of recommended IPM strategies resulting from this study is as follows:

1. The row spacing (or distance between the date palms) should not be less than 10 × 10 meters in order to allow sunlight penetration and wind movements between the palms.
2. The offshoots should be separated from the mother palms at the proper age because their presence around the parent palm increases the level of infestation.
3. Reduce or stop irrigation of the date palms for at least 4 to 7 weeks before DB generation (spring and autumn) each year. This is vital to reduce the juices on the frond which most likely contribute to DB survival.
4. Insecticides should be used with precise scientific implementation based on other measures such as GIS and remote sensing.
5. Establish a GIS database to improve the main processes for date palm cultivation such as propagation, irrigation, pruning, pollination, fruit thinning, fertilisation, and pest control.
6. Educating farmers and offering refresher courses for extension service personnel and crop protection officers.

## Supporting information

S1 File Blank survey. (PDF)
