# Supplementary material for: Impacts of human-related practices on Ommatissus lybicus infestations of date palm in Oman
Source: PLoS One. 2017 Feb 6;12(2):e0171103. doi: 10.1371/journal.pone.0171103 (PMC5293203; doi:10.1371/journal.pone.0171103)
Supplement: S1 File — (PDF) [file pone.0171103.s001.pdf]

Field work questionnaire

"An analysis of the impacts of human-related practices on Dubas bug infestations on date palms in northern Oman using Remote Sensing and GIS"

Please note

For this survey:

This questionnaire should be completed by or on behalf of farmers who operate their own farm, work of farmers on rented farm or land farmed on shares.

1. Please indicate the ownership of the farming unit. Is the unit owned by : (mark with X)

☐ Individual	☐ Close corporation	☐ Trust
☐ Family	☐ Government enterprise	☐ Partnership
☐ Private company	☐ Public corporation	☐ Public company

Other (specify)

☐

2. Particulars of the principal farmer / farm operator
   a. Surname and name …………………………………………………………………….
   b. Governorate ……………………District…………………Village name……………...
   c. Age……………………………Education…………………………………………….
   d. Farm area ……………………………………………………………………………
   e. Telephone or cellphone number……………………………………………………..
   f. Fax number ……………………………..Email address………………………………

3. Number of owners/ family workers
   a. Owner directly involved in farming activities
   b. Family members involved in farming operations who do not receive regular salaries

	Males	Females
a.		
b.		

4. Number of full employees who receives regular salaries
   a. Employees who normally do farm work

	Males	Females
a.		

Nationality (Specify) ………………………………….

Please complete the table and mark with (x)

ID	Water and Irrigation		Yes always	sometimes	No	Don't know
1		Is water available to irrigate the farm?				
2		Are the water resources protected from human and animal contaminations?				
3		Is there a specific a type of palm tree at needs a high amount of water in order to grow?				
4		Is water used in irrigation salty?				
5		Is the dump tank water properly maintained and cleaned?				
6		Does the Government provide irrigation systems for the farm?				
7		Do you feel that the irrigation systems protect your fame from the weeds?				
8		Is surface water (falj, river or wadi) the main source for irrigation in the farm?				
9		Is groundwater the main source for irrigation in the farm?				
10		Is the water source protected from run-off and flooding?				
11		Do you have multiple sources to irrigate the farm?				

Do you have any further points?

………
………
………

Please complete the table and mark with (x)

ID	Questionnaire		Yes always	sometimes	No	Don't know
1	Date palm propagation Practices	Do you have a specific time (stage) in transplanting the palm trees?				
2		Do you choose one season for planting palm trees?				
3		Do you have a specified process for germinating palm trees?				
4		Do you have a designated time of the year for germinating palm trees?				
5		Do you have a regular separation of seedlings?				

ID	Field Work Questionnaire		Yes always	sometimes	No	Don't know
1	Physical Location and farm	Do topographical features (slope, depressions, etc.) exist near the field that might encourage run-off?				
2		Is an optimal distance between the palm trees and potential contaminants: septic, drain fields, chemical storage, gas tanks, manure piles, etc.?				
3		Are alfalfas or clovers grown on the same farm?				

Do you have any further points?

………
………
………
………
………

Please complete the table and mark with (x)

ID	Field Work Questionnaire (Dubas Bug)		Yes always	sometimes	No	Don't know
1	Pests	Are seedlings examined for pests before planting?				
2		Are pests properly controlled according to a schedule?				
3		Do you use ground pesticides to kill pests in the farm?				
4		Do you know about the Dubas bug insect and its impact on the palm trees and productions?				
5		Does the Government provide both ground and aerial spray for pests?				

5. Please answer questions
   a. When the last aerial spray at the years in the farm?
      ………………………………………………………………………………………
      ………………………………………………………………………………………
      ………………………………………………………………………………………
      ………………………………………………………………………………………
   b. What kind of pesticides regularly use for pests in the farm?
      ………………………………………………………………………………………
      ………………………………………………………………………………………
      ………………………………………………………………………………………
      ……………………………………………………………………………………...

Do you have any further points?

……
……
……
……
……
……
……
……

ID	Field Work Questionnaire		Yes always	sometimes	No	Don't know
1	Worker management	Are standard operating procedures in the place for clearing farms in post-harvest operations?				
2		Do employees feel responsible for palm tree safety and the dates as well?				
3		Are employees with illness and open wounds prohibited from handing produce?				
4		Is farm equipment cleaned and sanitized routinely?				
5		Are training schedules, sanitation policies, and personal hygiene policies documented and accessible to employees?				
6		Are there standard ways to keep your farms clean from wastes?				
7		Do you regularly remove weeds farm under palm trees?				
8		Do you regularly remove the old and non-economic palm trees from the farm?				
10		Do you remove fruit fallen and from the palms after harvesting directly?				
11		Is there a space of least (8X8) meters between palm trees?				
13		Is there a standard depth of planting palm trees?				

6. Person should contact if any queries arise regarding the complete questionnaire
   I. Name………………………………………………………………………………...
   II. Telephone number……………………………………………………………...
   III. Position/ title …………………………………………………………………....
   IV. Date ………………………………………………………………............................
   V. Email address…………………………………………………………………..
   VI. Signature …………………………………………………………………………….
